# The mediating role of organizational intelligence in the relationship between quantum leadership and innovative behavior

**DOI:** 10.3389/fpsyg.2022.1051028

**Published:** 2022-10-26

**Authors:** Ayşe Bilgen, Meral Elçi

**Affiliations:** Department of Business Administration, Social Sciences Institute, Gebze Technical University, Kocaeli, Turkey

**Keywords:** innovative behavior, quantum leadership, organizational intelligence, leadership, COVID-19 pandemic

## Abstract

The present study aims to examine the mediator effect of organizational intelligence between quantum leadership and innovative behavior of employees in health organizations. It is aimed to examine the mediator effect of organizational intelligence between quantum leadership and innovative behavior of employees in health organizations. The data of the study were collected from 626 healthcare professionals working in hospitals and health centers in Istanbul, Turkey, by survey method. After the analysis of normality, validity and reliability, the hypotheses of the research were tested on the LISREL program using the structural equal modelling. The findings have showed that the three hypotheses of the research were confirmed: quantum leadership and organizational intelligence affect the innovative behaviors of employees positively and significantly, and organizational intelligence has a mediator effect between quantum leadership and the innovative behavior of employees. It is imperative that healthcare organizations, which are likely to encounter chaos and crisis risks such as the Covid-19 pandemic, follow innovation in order to provide better quality services. Quantum leadership and organizational intelligence are necessities in terms of revealing innovative behavior for healthcare organizations operating in a dynamic and chaotic environment. Since the effects of quantum leadership have not been studied in health organizations adequately, this study makes a contribution to fill this gap. In addition, it is predicted that the findings of this research will be illuminating in terms of innovative behaviors and leadership styles for health organizations that have a vital importance in all societies and have recently experienced a fact like COVID-19.

## Introduction

Today, there are many factors that determine the conditions of working life. While globalization, technological developments, changes in people’s expectations, unexpected chaos and crisis necessitate change and innovation; moreover, these factors complicate the management of working life conditions. Both benefiting from the advantages of these challenging conditions and being prepared for the risks of these conditions are related to the openness of organization and its employees to innovation, having a high level of organizational intelligence, and having abilities to exhibit effective leadership even in chaos and crisis.

Health organizations carry out a very vital task that affects human life, health, quality of life and therefore happiness. Health organizations are also affected by the intense changes experienced nowadays (Dodson, 2017). It is a necessity for health organizations to adopt these changes in accordance with the changing socio-cultural values, and reflect these values to their customers. For these reasons, it has become a necessity for health workers to be in a constant change in order to catch the dynamism in health services (Jasper, 2005). Due to the sensitivity of the task carried out by health organizations, even little error might not be tolerated. Therefore, health organizations have to both change and not make mistakes while changing (Manion, 1993). As a result, health organizations are expected to have high skills and competencies and have highly talented leaders.

Compared to the past, more emphasis is placed on the continuous learning and innovative behavior of employees today, and employees are encouraged to exhibit innovative behaviors (Shelton and Darling, 2003; Chowdhury, 2005). Organizations support the innovative behaviors of their employees in order to increase their operational efficiency, provide better service to the customer, and increase the competitiveness and performance of organizations (Sigala, 2012; Obeng and Boachie, 2018). It is posited that the needs and expectations of customers will be met at a higher level in organizations that have employees with high levels of innovative behavior (Li and Hsu, 2018).

However, there is always the possibility that the innovations will affect the existing system and practices negatively (Ford, 1996). For this reason, organizational managers have a critical role in both encouraging and effectively managing the innovative behaviors of employees (Lindsey and Mitchell, 2012). Today’s complex, uncertain and rapidly changing working life makes it difficult for leaders (Dodson, 2017). The Covid-19 pandemic, which created an environment of crisis and chaos in all sectors, especially the health sector, has led to the questioning of classical leadership styles (Forster et al., 2020; Ortega and Orsini, 2020; Stefan and Nazarov, 2020). There is a need for more modern leadership styles, such as quantum leadership, that can cope with today’s challenging conditions. Looking at health care from the perspective of quantum theories offers new perspectives on management techniques for the effective and efficient delivery of health care (McDaniel, 1997). Since the quantum leadership style foresees a constant change in organizations in terms of place, time and people, it will enable the formation of a wide variety of information, communication and interaction networks (Chadwick, 2010; Curtin, 2011). In this respect, benefits such as the development of multifaceted relationships and the recognition of each individual as a leader through teamwork, cooperation and communication in organizations will be possible (Değirmenci and Utku, 2000; Uzunçarşılı, 2002; Grossman and Ve Valiga, 2005; Colón-Emeric et al., 2006). Thus, each employee will be able to see her or his own potential, open new horizons for herself or himself, and achieve better outcomes by improving her or his skills instead of following others (Kara, 2013).

Another important concept for organizations in today’s challenging conditions is organizational intelligence. Organizational intelligence, which is related to the collective use of all the skills of the organization, is one of the factors that determine the efficiency, performance, competitiveness and success of an organization (Liang, 2001; Schwaninger, 2001; Al Shobaki et al., 2017). Organizational intelligence, which can be defined as the whole of the abilities that make the existence and sustainability of an organization possible and the use of these abilities (Erçetin, 2001), is closely related to the innovative behavior levels of the employees in this respect. In the definition of a collective combination of all intelligences that contribute to the shared vision, renewal processes and direction determination within the organization (Liebowitz, 1999), the relationship between organizational intelligence and innovative behavior is shown, as well. Since the conditions of the management environment of the leaders, who are responsible for managing resources of organizations in the most efficient way, are determined by the level of organizational intelligence, it can be said that there is a reciprocal relationship between leadership and organizational intelligence (Keleş and Özkan, 2010; Turan and Erçetin, 2017). Quantum leadership’s features suitable for today’s conditions are such as to provide the appropriate environment for the protection and development of organizational intelligence (Morrison, 2002; Akçakaya, 2010).

In this research, it is aimed to examine the mediator effect of organizational intelligence between quantum leadership and innovative behaviors of employees in health organizations. Previous researches have emphasized organizational intelligence affects processes such as performance and innovation orientation considering the environmental conditions, organizational types and organizational capacity (Akgun et al., 2007). In addition, it has been stated that leaders who create change in organizations motivate their subordinates to do more, that is, to increase their potential (Burke and Collins, 2001). Knowing the sources of motivation on subordinates is among the general characteristics of quantum leaders in particular (Penrose, 1999). It is evident that there is a need to investigate the relationship between innovative behaviors, quantum leadership and organizational intelligence. Since the effect of quantum leadership on innovative behavior has not been sufficiently studied in health organizations, this study makes a contribution to fill this gap. In addition, it is predicted that the research findings will be illuminating in terms of innovative behaviors and leadership styles for health organizations that have a chaotic experience like the COVID-19 pandemic in all societies. The findings of this research will guide practitioners to increase the quality of service in health organizations, which provide a vital service to increase the quality of life of people, to regulate the functioning of health organizations and to increase the performance. The findings will help to raise awareness of the innovative behavior and organizational intelligence of employees in health organizations and will lead health organizations to implement a new and contemporary leadership style, quantum leadership.

## Literature and theoretical background

### Innovative behavior

Innovative behavior means creating, introducing and applying new ideas about increasing the performance of the job or group (Orfila-Sintes and Mattsson, 2009). From an organizational point of view, innovative behavior is defined as individual activities that occur in an organization operating in any sector, in the form of developing and adopting an innovation that benefits the entire organization, and putting that innovation into action by applying it in the organization (West and Farr, 1989). Innovative approaches in terms of change and creativity are important in resolving events faster and managing processes more successfully (Scott and Bruce, 1994). The continuous self-renewal and development of an organization and therefore its sustainable success depends on the innovative behavior of employees (Park et al., 2014).

Innovative behavior in the workplace is a complex process consisting of different stages. According to Janssen (2000), the completion of this behavior usually consists of three stages. These are idea generation, idea promotion, and idea realization. Idea generation, beginning stage of innovative behaviors in the workplace, means the generation of new and useful ideas in a work-related field. Work-related problems, incompatibilities, discontinuities and different trends can be triggers for the idea generation stage to begin. The idea generation stage is followed by the idea promotion stage in the sense of transferring the new idea produced to others. When an employee comes up with a new idea in the workplace, he or she tries to generate the necessary power and support for the idea to come to life by transferring it to his or her friends, supporters and supporters who are likely to support this idea. The last stage of innovative behavior in the workplace is idea realization. The implementation of the idea that was produced and spread is started at this stage. The implementation of the new ideas produced may be by a single person or by the entire organization. It may be possible to realize simple innovations through individual efforts. More complex innovations may require special qualifications, knowledge, role distribution and teamwork.

Creativity has an important and triggering effect on the emergence of innovative behavior. It has been suggested that innovative behavior is a situation related to motivation as well as knowledge and ability. Employees who have the capacity to display innovative behavior may need a stimulus to use this capacity. In this respect, it has been stated that the motivational side of innovative behavior paves the way for innovative behavior to take place in leadership research (Pieterse et al., 2010).

With the development of science and technology, one of the areas where changes are experienced most frequently and necessarily is health. For this reason, it is a necessity for healthcare professionals to show innovative behavior and be open to innovation (Jasper, 2005; Dodson, 2017). However, it is necessary to be very meticulous and careful about innovations in the field of health because the functions in the field of health are related to human health and are linked to some basic procedures (Manion, 1993). A small change in the implementation of health services can have a large number of consequences (Haigh, 2002). Additionally, there are specific behavioral patterns that emerge over time in groups of clinical staff, and that each employee has a set of rules guiding their behavior (McDaniel and Driebe, 2001; Litaker et al., 2006). These features show the complexity of health services. This complexity necessitates the management of innovative behaviors of healthcare workers.

### Quantum leadership

Leadership is one of the most frequently and extensively discussed concepts. The characteristics of the ideal leader and the appropriate leadership style for organizations have always been the subject of discussion (Kruger, 2009). Leadership characteristics and leadership styles has always differed according to the conditions of the period. The introduction of the Quantum Theory at the beginning of the 20th century caused radical changes in the field of physics. This effect of change has inspired other fields as well. This effect has also been seen in the field of management and the quantum leadership view has been developed.

Quantum leadership is a new paradigm, and it is envisaged that this new leadership style will bring organizations from the 20th century industrial age to the 21st century information and technology age and gain them skills and behaviors that will take them further. In terms of this understanding, the ultimate goal of organizations is the climax, which can be called the chaos limit. The most appropriate words to describe the field of activity of quantum leadership are uncertainty and discontinuity, as well as disorder and confusion. In chaotic environments and the management of these environments, the first and main effect is determined by the attitudes and behaviors of the leaders. It is stated that regular leaders mostly lose their ability to think proactively due to anxiety and fear in crisis environments, and cannot recognize systems that are environmentally sensitive, primarily because they resist change and different perspectives (Cooper and Sawaf, 1997). In this context, management skills are most needed in crisis situations. In such uncertain chaotic situations, some classical management theories lose their functions. Although the effectiveness of different leadership styles in the management of chaotic environments is mentioned, it can be accepted that the effect of quantum leadership is higher in such environments (Hanine and Nita, 2019).

Before quantum theory, the perceived universe was a uniform, simple, predictable and precise universe with absolute reality and absolute perspective. On the other hand, the universe predicted by Quantum Theory is a probabilistic, unpredictable, pluralistic, diverse, uncertain and complex universe. In this sense, it has been argued that because there is pluralism, diversity, uncertainties and confusion in organizations as predicted by Quantum Theory, the type of leadership compatible with these structures of organizations is quantum leadership (Fris and Lazaridou, 2006).

Change in the concept of quantum leadership is a normal phenomenon and cannot be prevented. Individuals cannot avoid change, but can learn how to manage it. Thus, they can successfully adapt a structured and effective change to their organizations (Colón-Emeric et al., 2006; Hast et al., 2013). Especially unexpected and sudden changes bring along chaotic environments. It is necessary to carry out strategic studies with increased effectiveness and to develop a leadership approach suitable for process management in chaotic environments. Managing chaotic environments requires an interactive perspective from leaders. Both the quantum leadership’s ability to manage chaotic, uncertain and complex environments and the importance it attaches to the interaction of the leader and the managed make this leadership style a suitable option for the management of chaotic environments (Uhl-Bien et al., 2007).

Quantum leaders are expected to try multiple activities in practice, to care about the informal relationships that shape the thoughts and actions of the team, and to maintain the balance between cooperation and competition, rather than the understanding of “one must be sure to do something” (Burns, 2001). It is posited that when the team is in a difficult situation or when change is needed, quantum leaders eliminate resistance, evaluate whether employees participate in activities, and support individuals to reveal their competitive side (Chadwick, 2010).

### Organizational intelligence

Organizational intelligence, which is the ability of an organization to produce information and use information strategically, is one of the organizational capabilities that can affect success of an organization (McMaster, 1996; Halal, 2006). Organizational intelligence is a continuous cycle of activities that includes perceiving the environment, producing meaning through developing and interpreting perceptions, using memory of past experiences to assist perception, and taking action (Choo, 1995). Organizational intelligence helps to identify the weak sides of an organization and to strengthen the constructive sides. Organizational intelligence, which is based on human intelligence, is characterized as a continuous thought capital that can increase the flexibility of organizations if applied and managed correctly. In addition, it also promotes the development of creativity and innovation power by accelerating knowledge acquisition, knowledge management and organizational learning (Gholami and Safaee, 2012).

Organizational intelligence expresses the existence of the concept of intelligence for the whole organization rather than measuring the individual intelligence of members within a group. This makes organizational intelligence a social output that helps to evaluate an organization in general rather than the sum of individual intelligences (Glynn, 1996; Stalinksi, 2004). In this respect, organizational intelligence can be characterized by the ability to make sense of complex situations and act effectively, which are accepted as collective skills, to interpret and act according to the events and signs in the environment, to produce, use and share information appropriate for the purpose, and to learn and reflect from experience (Veryard, 2018).

Organizational intelligence is one of the factors that increase the solving capacity of the organization and organizational intelligence consists of more than one cognitive subsystem (Halal, 2006). These systems are: organizational structure (the structure that has authority over which decisions to be made), organizational culture (the values and rules that guide actions), stakeholder relations (what amount of information is exchanged among different groups), knowledge management (the type and amount of valid information), and strategic processes (how knowledge is channeled into understanding, learning and action). All these subsystems serve as fundamental decisions in the cognitive functions of the organization and collectively create organizational intelligence. The five subsystems of organizational intelligence can be considered as the intellectual strength of an organization. Organizational intelligence shows an impact activity that focuses on eliminating the complexity of the working activities of organizations in the general context. Thus, studies showing that organizations are enabled to be functional at an effective level point to the support and need for organizational intelligence (Liang, 2001).

In today’s world where expectations differ, organizations need more new skills and competencies to be successful and to ensure sustainability. One of these skills and competencies is organizational intelligence. Human resources can be used in the most efficient way and satisfactory results can be obtained with the effective management of organizational intelligence (Falletta and Combs, 2018). On the basis of the systematic development and improvement of many processes, the effectiveness of organizational intelligence and the management of such factors are evaluated. In this context, organizational intelligence and its consequences have features that can explain the success of an organization. Developing activities that will contribute to organizations gaining competitive advantages offers a potential to achieve significant results by adhering to the interactions between the organization and the environment (Al Shobaki et al., 2017). Organizational intelligence helps organizations speed up processes, rationalize processes, increase quality and develop skills. In addition, organizational intelligence contributes to the development of the society in which organizations operates. Moreover, the ability of individuals and the use of their skills at a competent level are provided with the importance given to organizational intelligence for the development of organizations (Schwaninger, 2001). For this reason, the answers to the questions “how can organizations be smarter” and “how can a high level of intelligence be maintained in organizations” have gained importance today.

## Research hypothesis

**Figure 1:**
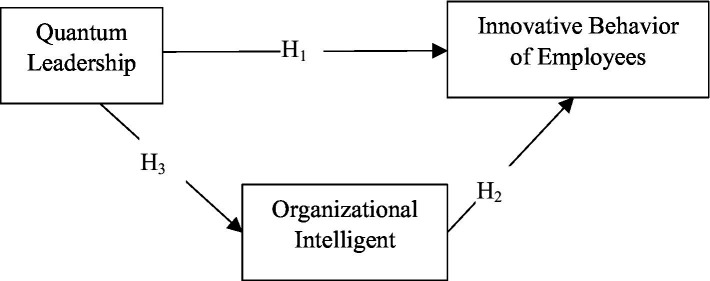
Research model.

In accordance with the research model shown in Figure 1, three hypotheses were defined between the innovative behavior of employees, quantum leadership and organizational intelligence variables.

### The effect of quantum leadership on innovative behavior

Determining new goals and strategies for the organization according to the changing conditions, being a change agent for the organization, and ensuring the success of the organization under all conditions by adapting the organization to the changing conditions are among the primary duties of the leaders (Kruger, 2009). Supporting employees by leaders is accepted as a factor affecting innovative behaviors (Parker et al., 2006; Hunter and Cushenbery, 2011). It is possible for employees to generate creative ideas, create new opportunities and turn the created opportunities into useful actions, thanks to the leaders creating an environment in which there is a qualified communication between employees in organizations, effective feedback is provided, and everyone can freely express their opinions (Prieto and Perez-Santana, 2014). In this context, it is critical for organizations to have a leadership style that will understand changes, manage them and prepare organizations for these changes (Scott and Bruce, 1994; Naktiyok, 2009).

It is argued that the quantum leadership style has the potential to meet this need of organizations, and that this leadership style forms the basis for the new way of thinking of the scientific theories that have emerged recently (Shelton and Darling, 2003). Quantum leadership is suitable for those who want to create learning organizations and create a new science-based skill set that enables twenty-first century leaders to do much more than just organizational adaptation to proactive change and continuous learning (Hanine and Nita, 2019).

One of the prominent features of quantum leadership is that experimental thinking, change and development can be initiated from any part of the organization (Fris and Lazaridou, 2006). This feature means that quantum leaders and the organizations they manage are ready for change from anywhere in the organization at any time.

Learning organizations cannot simply be created by leaders who operate under the traditional, mechanical organizational paradigm. If leaders are to create original learning organizations, they need to adapt a new perspective, a new paradigm or mental model to reality (Shelton and Darling, 2003). At this point, new scientific theories of quantum mechanics lay the foundation for a new way of thinking about organizations.

Quantum leadership attaches importance to communicating with employees, cooperating with them, valuing their ideas and supporting them by conducting informal relations when necessary (Burns, 2001). Employees who realize that their leaders are listened to and their opinions are taken into consideration will increase their organizational commitment and increase their productivity (West et al., 2015).

In the work environment created by quantum leadership, employees will be able to consider emerging problems as an opportunity, use their creativity and develop new solution proposals. With the understanding of flexibility provided by quantum leaders, the current situation in the system will disappear and the change will be able to take its place in the systems of the organization. Accordingly, leaders should encourage individuals so that each employee can carry out their activities independently, integrate the individual visions of the employees and the strategic goals of the organization on a common denominator, and evaluate the performance of individuals with the shared values created (Curtin, 2011).

Studies in the literature also confirm the positive relationship between quantum leadership style and innovative behaviors of employees. It has been found that quantum leadership has a positive and significant effect on organizational innovation (Ahang et al., 2020). It has been reported that as the use of quantum skills in management increases, organizations see, think, feel, know, trust and act in a way that enables their employees to become proactive change agents (Shelton and Darling, 2003). A positive and significant relationship was also found between teachers’ quantum skills and creativity (Sardashti and Pordanjani, 2019). Based on the above discussions, the H1 hypothesis of the research was developed:

*H 1*: Quantum leadership has a positive effect on innovative behavior levels of employees.

### The effect of organizational intelligence on innovative behavior

Organizational intelligence has been defined above as having and using skills and competencies that affect the success and sustainability of the organization. Since market conditions and competition change and diversify rapidly, the skills and competencies that affect the success and sustainability of organizations also have to change and diversify. The innovative behavior competence of an organization and its employees is also decisive in acquiring the necessary skills and competencies according to changing conditions. It can be predicted that new skills and competencies required for the success and sustainability of an organization can be acquired more rapidly and comprehensively in organizations where the innovative behavior levels of their employees are high.

The actions of employees surround the practices that bring the intelligent organization understanding to the fore and thus enable faster and more precise decisions to be made. Organizations with high organizational intelligence can achieve successful results in adapting innovations to organizations and using their intuition (Erçetin, 2001). The creation of organizational intelligence means targeting organizational developments that will enable employees to think more creatively (Erçetin, 2004).

One of the sub-dimensions of organizational intelligence is knowledge management, and this concept requires timely learning and sharing and dissemination of the necessary information for an organization (Halal, 2006). It is clear that learning, sharing and disseminating new knowledge is closely related to the innovative behavior of employees. In this context, it can be predicted that the innovative behavior levels of employees will be high in organizations with high organizational intelligence that perform knowledge management effectively. Confirming this, many studies in the literature have suggested improving organizational intelligence as a prerequisite for the development of innovative behaviors (Nevis et al., 1995; Glynn, 1996; Teece et al., 1997; Romijn and Albaladejo, 2002).

Studies in the literature have also confirmed this relationship. In a study conducted in Iran, it was determined that there is a positive and significant relationship between organizational intelligence and employee creativity (Torabi et al., 2016). In another study, it was found that there is a positive and significant relationship between organizational intelligence and innovation management (Kahkha et al., 2015). Another study showed that organizational intelligence has a positive effect on willingness to innovate (Reza et al., 2014). Based on the above discussions, the H2 hypothesis of the research was developed:

*H 2*: Organizational intelligence has a positive effect on the innovative behavior levels of employees.

### The effect of quantum leadership on organizational intelligence

The role of leaders, who determine the goals and strategies of organizations, directs and supervises organizations, in acquiring the skills and competencies that organizations need for success and sustainability is indisputable. In this sense, the characteristics of leaders are accepted as a critical determinant on organizational intelligence (Torkamani and Maymand, 2016). In addition, organizational culture, which is one of the elements of organizational intelligence, is one of the effective factors that leaders use when determining the strategies they follow (Turan and Erçetin, 2017). Today, due to the abundance of complex and interactive systems, it requires the creation of a significant and high level of potential in organizations (Halal, 2006). Thus, organizational intelligence has a dimension that can transform organizations and assign important tasks to leaders.

The concept of knowledge management, which is defined among the subsystems that increase organizational intelligence, covers interconnected activities that include the creation, evaluation, storage, distribution and sharing of knowledge in order to provide the right information to the right person at the right time and at low cost through sharing and learning (Halal, 2006). Leaders, on the other hand, determine the strategic direction and vision of an organization regarding knowledge creation and sharing, provide motivation and coaching, and enable the creation of policies. In organizations with high organizational intelligence, leaders adopt a people-oriented management approach by placing people at the center. In such a management approach, employees are empowered and participate in management (Naktiyok, 2009).

Leaders must take knowledge out of his control and share it with all members of the organization. Effective leadership removes walls by distributing leadership throughout the organization. Ultimately, the momentum for growth and transformation comes from the entire organization, not just from the top. Because effective leadership is not to control from above, but to reveal the hidden power in people (Sullivan and Harper, 1997). In this context, quantum leadership is a type of leadership that includes features such as being process-oriented, creating a common vision, creating synergy and interaction, considering the organizational environment, cooperation, supporting the members of the organization, focusing on organizational and individual interests, seeing the members of the organization as a potential leader, being a learning leader, evaluating opportunities and creating opportunities (Morrison, 2002). In addition, the support that the quantum leader receives from the members of the organization stems from the trust, commitment and mutual respect (Akçakaya, 2010).

Today’s rapidly changing conditions, globalization, changes in values and differentiation of customers’ demands and expectations increase uncertainty for organizations and reduce predictability. It can be argued that organizations with quantum leadership are more likely to succeed in such chaotic environments. It is also seen that the characteristics of quantum leadership are more compatible with organizational intelligence. In the volatile and turbulent environment of today’s world, there is a need for talented leaders who will respond appropriately to every sudden change and event and show the necessary flexibility. Studies in the literature also support this prediction.

In a study examining the effect of school administrators’ quantum leadership behavior on the level of organizational intelligence, it was found that school principals’ quantum leadership behaviors had a positive significant relationship with the level of organizational intelligence (Turan and Erçetin, 2017). It was found in another study that there is a positive and significant relationship between the creativity of school administrators and organizational intelligence (Ordooie, 2016). In a study conducted at a university, it was found that quantum management skills positively affect organizational agility and organizational intelligence has a mediator effect on this effect (Salimi et al., 2019). Based on the discussions above, the *H3* hypothesis of the research was developed:

*H3*: Organizational intelligence has a mediating effect on the relationship between quantum leadership and innovative behavior levels of its employees.

## Research methodology

### Sample and procedures

The data of this research study were collected by questionnaire method. Questionnaires were conducted both face-to-face and online. The population of the research study consisted of health personnel working in the province of Istanbul in Turkey. The sample of the research consisted of 626 healthcare professionals working in different branches in the hospital and family health center who volunteered to participate in.

Demographic findings of the participants in the study are given in Table 1. According to the findings, 57.7% of the participants were female and 42.3% were male. A significant majority of the participants (40.1%) have bachelor degree. While the rate of those with a doctorate degree was 29.7%, the rate of those with a master’s degree was 17.4%. More than half of the participants were nurses (52.2%). While 22.3% of the participants were specialist doctors with different titles, 5.1% were general practitioners and 7.5% were assistant doctors. It was found that the ages of the participants ranged from 20 to 64, and the mean age was 37.0 (sd = 8.3).

**Table 1:** Demographics of the participants.

**Variable**		**n**	**%**
Gender	Female	361	57.7
	Male	255	42.3
Level of Education	High school	28	4.5
	Associate degree	52	8.3
	Bachelor Degree	251	40.1
	Master Degree	109	17.4
	Doctorate Degree	17	2.7
	Expertise in Medicine	169	27,0
Position	Assistant Dr.	47	7.5
	General Practitioner Dr.	32	5.1
	Specialist Dr.	100	16,0
	Associate Professor	27	4.3
	Prof. Dr.	13	2.1
	Nurse	327	52.2
	Other	80	3.8

### Measures

#### Innovative behavior scale

The innovative behavior scale was used to measure the innovative behavior levels of hospital employees. The scale was developed by Lukes and Stephen (2017) and adapted into Turkish by Pala and Turan (2020). The scale is a 5-point Likert-type scale consisting of seven dimensions and 23 items. In the scale, *1* corresponds to the expression *strongly disagree* and *5* corresponds to the expression *strongly agree*.

#### Quantum leadership scale

The quantum leadership scale was used to measure the quantum leadership behaviors of healthcare professionals. It was developed by Konan and Mermer (2021). The scale measures how managers are evaluated as a leader by their employees in terms of quantum leadership characteristics (example: my leader breaks traditional patterns, my leader knows how to deal with sudden negative situations). The scale is a 5-point Likert-type scale consisting of a single dimension and 24 items.

#### Organizational intelligent scale

The organizational intelligence scale was used to measure the perceptions of healthcare professionals about the organizational intelligence of the institution they work for. The scale was originally developed by Falletta (2008). The scale was adapted to Turkish by the researchers and the scale was revised by taking experts’ opinion. The scale is a 5-point Likert-type scale consisting of 11 dimensions and 52 items.

### Pilot study

Innovative behavior and Quantum leadership scales, which were decided to be used in the study, have already been used in Turkish culture. Organizational intelligent scale, on the other hand, has not been used in Turkish culture. Therefore, it was decided to use Confirmatory Factor Analysis (CFA) to determine whether this scale has a similar factor structure in Turkish culture, as well (Kline, 2011). No changes were made in the scale items and the number of items. CFA is performed to determine whether the factor structure of a scale determined *a priori* is preserved in a different culture (Hambleton and Patsula, 1999; Hambleton et al., 2005; Büyüköztürk et al., 2010). CFA is also used to design, review, define, and most importantly verify the relationships among variables related to the adaptation of a scale (Bentler and Bonett, 1980; Stes et al., 2010). It was assumed that it would be beneficial to conduct a pilot study to retest whether all scales used in the study including the ones adapted in Turkish culture already produce reliable results (Büyüköztürk et al., 2010).

In order to test the validity and reliability of the three scales used in the study, a pilot study was conducted with 136 healthcare professionals. The validity of the scales was tested by using confirmatory factor analysis (CFA) on the LISREL program. Model fit for all three scales was tested using CMIN, RMSEA, NFI, CFI, IFI, SRMR and AGFI model fit indices and it was seen that the model fit criteria were met. Factor loading scores were also found to be above 0.50. The reliability tests of the scales were made by calculating the Cronbach’s Alpha values. Cronbach’s Alpha values of all three scales were found to be above 0.90. In addition, it was found that the Corrected Item Total Correlation values of all items in the scales were above 0.30. Since the validity and reliability scores obtained from the pilot study related to the three scales used in the research study were acceptable, they were used in the main study without making any changes.

### Analytical strategy

SPSS and LISREL programs were used to analyze the data. The analyzes of descriptive statistics and reliability analyzes of the scales were conducted on the SPSS program. The confirmatory factor analysis (CFA), which was conducted to test the validity of the scales, and the testing of the structural model of the research using structural equation modeling (SEM) were carried out on the LISREL program.

### Ethical considerations

Ethical clearance to conduct this study was obtained from the Gebze Technical University Research Ethics Committee (official document number 2021/28–02). This article followed all ethical standards for research.

## Results

### Validity and reliability

The validity of the three scales used in the study was tested by using a CFA. According to the CMIN, RMSEA, NFI, CFI, IFI, SRMR and AGFI model fit index findings (Table 2), the CFA models showed an acceptable fit. In addition, all factor loadings scores were found to be higher than 0.30. In this study, the lowest factor loading score in the innovative behavior scale was 0.59, while the highest was 0.87. The lowest factor loading score in the quantum leadership scale was 0.63, while the highest was 0.81. Finally, the lowest factor loading score in the organizational intelligence scale was 0.38, while the highest was 0.90. In addition, correlations between all path coefficients and latent variables were found to be significant (*p* < 0.05). Perfect and acceptable fit indices according to Bollen (1989), Tabachnick and Fidell (1989), and Hooper et al. (2008) presented in Table 2.

**Table 2:** DFA model fit.

**Index**	**Perfect fit**	**Acceptable fit**	**Innovative behavior**	**Quantum leadership**	**Organizational intelligent**
CMIN	< 2,00	< 3,00	2,92	4,61	4,45
SRMR	< 0,05	< 0,10	0,04	0,04	0,06
NFI	> 0,95	> 0,90	0,98	0,98	0,98
CFI	> 0,95	> 0,90	0,99	0,98	0,98
IFI	> 0,95	> 0,90	0,99	0,98	0,98
AGFI	> 0,90	> 0,85	0,91	0,87	0,83
RMSEA	< 0,05	< 0,10	0,06	0,08	0,07

In this study, there are 99 reflective indicators in the measurement model and three latent factors corresponding to the three constructs. The measurement model was examined using convergent, divergent and discriminant validity (Table 3). Cronbach Alpha values were calculated to test the reliability of the scale. All of the CR coefficients of the scale were above 0.75. AVE values were above 0.50 except for the quantum leadership scale (0.44). Although it is recommended that the AVE value be 0.50 and above, if the BG coefficient is 0.70 and above, an AVE value of 0.40 and above is considered acceptable (Fornell and Larcker, 1981; Peterson, 2000). Fornell and Larcker (1981) criteria were used for discriminant validity. Accordingly, the correlation coefficients between the square root of the AVE value and each structure in each row-column were examined, and seen that the correlation between each construct was below the square root of the AVE value. Therefore, it was accepted that each structure measures a different feature from the other. The scales were assumed as reliable because all of the scales had Cronbach’s Alpha values above 0.90 (Nunnally, 1978).

**Table 3:** Cronbach’s Alpha, AVE and CR values for each measures and correlations.

**Measures**	**Cronbach’s Alpha**	**CR**	**AVE**	**1**	**2**	**3**
1-Quantum Leadership	0,96	0.88	0.44	(0.66)		
2-Organizational Intelligent	0,95	0.84	0.51	0.50	(0.71)	
3-Innovative Behavior	0,94	0,76	0,56	0,51	0.40	(0.75)

CR = Composite Reliability, AVE = Average Variance Extracted. Square roots of average variances extracted are shown on diagonal.

### Descriptive statistics, normality, and correlations

The findings related to descriptive statistics, normality and correlations are given in Table 4. Innovative behavior level with an average value of 4.05 and quantum leadership level with an average value of 3.94 were relatively higher than organizational intelligence with an average value of 3.55. Since the skewness and kurtosis values were in the range of ±1, the distribution of the data was considered to be normal (George and Mallery, 2010). Correlation analyzes showed that there is a positive and significant relationship between innovative behavior, quantum leadership and organizational intelligence (*r* = 0.48 and *p* < 0.05, *r* = 0.40 and *p* < 0.05, respectively). In addition, a positive and significant relationship was found between quantum leadership and organizational intelligence (*r* = 0.49 and *p* < 0.05).

**Table 4:** Descriptive statistics, Test of normality and correlations.

**Scale**	**N**	**Mean**	**Sd**	**Skewness**	**Kurtosis**	**1**	**2**	**3**
1-Quantum Leader	626	3.94	0.79	−0.26	0.13	1		
2-Organizational Intelligent	626	3.55	0.84	−0.49	−0.41	0.49^*^	1	
3-Innovative Behavior	626	4.05	0.63	−0.64	0.54	0.48^*^	0.40^*^	1

* *p < 0.01.*

### Hypotheses testing

**Figure 2:**
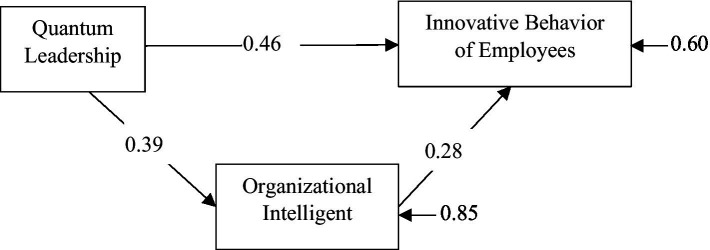
Result of LISREL path analysis (*n* = 626).

After the analysis of the measurement model, the model presented in Figure 1 was analyzed using path analysis to test the research hypotheses. The results of the path analysis are presented in Figure 2. As hypothesized, it was found that quantum leadership (*β* = 0.46 and *p* < 0.05) and organizational intelligence (*β* = 0.28 and *p* < 0.05) had a positive significant effect on innovative behavior. It is also found that quantum leadership has a positive significant effect on organizational intelligence (*β* = 0.39 and *p* < 0.05).

The model was saturated and the fit was perfect. In the analysis results, Chi-Square = 0.0; Degrees of Freedom (df) =0.0 and RMSEA = 0.00 values were found. Also, other indexes are CMIN = 1; SRMR = 1; NFI = 1; CFI = 1; IFI = 1; AGFI = 1 were found. Path analysis was performed using LISREL to examine the mediation and total effects of organizational intelligence (Joreskog and Sorbom, 1989) and the findings are given in Table 5. According to the findings, both quantum leadership and organizational intelligent had a significant effect on innovative behavior of employees. Quantum leadership significantly predicted organizational intelligent. In addition, the mediation role of the organizational intelligent was significant. Thus, all hypotheses in the research were supported.

**Table 5:** Total and indirect effects.

	**Total effect**		**Indirect effect**
	**Quantum leadership**	**Organizational intelligent**	**Quantum leadership**
Innovative Behavior	0.63 (0.04) 17.46	0.39 (0.04) 10.61	0.12 (0.02) 6.59
Organizational Intelligent	0.39 (0.04) 10.61	0.31 (0.04) 8.40	

## Discussion and conclusion

Health organizations carry out a critical task that affects human life itself and the quality of life. In order for this critical task to be carried out in a dignified and satisfactory manner, changes in the field of health must be implemented quickly and without any error. Successful realization of this vital change requires high organizational intelligence levels of health organizations and high innovative behavior levels of health workers (Manion, 1993; Liang, 2001; Schwaninger, 2001; Jasper, 2005). On the other hand, the existence of scenarios that disrupt and shake all systems such as the Covid-19 pandemic, especially in terms of health organizations, reveals the need for modern leaders who can effectively manage health organizations in variable, uncertain and complex environments. It is necessary for these leaders to be able to successfully manage health organizations by influencing organizational intelligence and innovative behavior levels of employees, even in unpredictable chaotic environments. Since it is assumed that quantum leadership, which has emerged in recent years, has such a potential (Fris and Lazaridou, 2006; Hanine and Nita, 2019; Tsao and Laszlo, 2019), this study examined the direct and indirect effects of quantum leadership and organizational intelligence on the innovative behaviors of employees in health organizations.

Research findings have showed that healthcare professionals have high levels of innovative behavior and perceptions of quantum leadership in their organizations. However, organizational intelligence levels were found to be relatively low. All three hypotheses of the study were confirmed. First, the findings have showed that quantum leadership positively affects the innovative behavior of employees in health organizations, which is consistent with previous studies (Shelton and Darling, 2003; Ahang et al., 2020). Second, the findings have showed that organizational intelligence positively affects the innovative behaviors of employees in health organizations, which is consistent with previous studies (Reza et al., 2014; Kahkha et al., 2015; Torabi et al., 2016). Third, quantum leadership in health organizations positively affects organizational intelligence, which is consistent with previous studies (Turan and Erçetin, 2017; Salimi et al., 2019). In addition, the findings have showed that quantum leadership in health organizations indirectly affects the innovative behavior levels of employees through organizational intelligence.

Based on the findings of this research study, it can be suggested to develop practices to increase the innovative behavior levels of the employees in health organizations where a chaotic environment is generally dominant and changes are experienced in order to provide up-to-date and more effective health services. While selecting personnel, it can be aimed to improve the level of innovative behavior with various training programs in which up-to-date information and technological developments about the field of activity are processed, as well as the selection of candidates with a high level of innovative behavior.

According to the findings, since the practices that will increase the organizational intelligence will increase the innovative behavior levels of the employees, the implementations to increase the organizational intelligence should also be planned by the organizations. In this context, individual intelligence can be strengthened with personal development or training programs that will make the existing potentials of employees who are critical for organizations functional, such as self-awareness, motivation, empathy and mastery in relationships. In addition, the interaction and communication of the organization with its environment, the use of new technologies and systems, performance evaluation and rewards, employee participation, taking decisions that will minimize resistance to change, informing the personnel about the organizational strategies, and determining the interaction and communication channels within the organization might be useful.

Since it has been found that quantum leadership positively affects both innovative behavior levels and organizational intelligence, applications should be developed that will ensure the dissemination of quantum leadership in health organizations. In this context, the participation of leading managers in professional development activities organized for health organizations and the creation of opportunities to take decisions together with employees in this direction can be given as an example in the context of strengthening the leader-follower interaction. When choosing managers, it may be considered to prefer managers who show quantum leadership characteristics that encourage learning, support their subordinates with their superior abilities and efforts, encourage their subordinates and follow strategies to increase their motivation, and can develop an understanding that focuses on individual development. In addition, training programs should be developed for existing managers in the health field to redefine leadership and to enable them to acquire the characteristics of quantum leadership, which offers an experiential learning method based on quantum leadership theory.

In this research study, a subject that has been rarely studied in the literature was investigated and important findings were obtained. A perspective has been provided on the changes in health organizations in times of crisis and chaos and the position of health workers in these changes. In addition, it has been pointed out that the ability of health organizations to give appropriate responses in times of crisis and chaos depends on their organizational intelligence level, and the mediator effect of organizational intelligence level on the innovative behaviors of employees was shown. Finally, it has been showed that quantum leadership affects the innovative behavior of employees both directly and indirectly through organizational intelligence.

However, the research has some limitations. The data of the study were collected from the participants selected by a non-probabilistic sampling method only from health institutions in the province of Istanbul in Turkey. Although the sample size is satisfactory, this feature of the sample can be considered as a limitation in terms of representativeness. The findings of the study should be interpreted with this limitation in mind.

Future research should more frequently examine the innovative behaviors of employees in healthcare organizations and the impact of quantum leadership and organizational intelligence on these behaviors. Additionally, qualitative researches and mixed method researches should be conducted to obtain a better and deep understanding of the relationships between innovative behaviors, quantum leadership and organizational intelligence.

## Data availability statement

The raw data supporting the conclusions of this article will be made available by the authors, without undue reservation.

## Ethics statement

The studies involving human participants were reviewed and approved by Gebze Technical University, Human Research Ethics Committee Decision (2021/28–02). The patients/participants provided their written informed consent to participate in this study. Written informed consent was obtained from the individual(s) for the publication of any potentially identifiable images or data included in this article.

## Author contributions

All authors listed have made a substantial, direct, and intellectual contribution to the work and approved it for publication.

## Conflict of interest

The authors declare that the research was conducted in the absence of any commercial or financial relationships that could be construed as a potential conflict of interest.

## Publisher’s note

All claims expressed in this article are solely those of the authors and do not necessarily represent those of their affiliated organizations, or those of the publisher, the editors and the reviewers. Any product that may be evaluated in this article, or claim that may be made by its manufacturer, is not guaranteed or endorsed by the publisher.
